# Specific Therapeutics

**Published:** 1909-01-09

**Authors:** 


### The Hospital

A Journal of

The Medical Sciences and Hospital Administration.

New Series. No. 97, Vol. IV. [No. 1169, Vol. XLV.] SATURDAY, JANUARY 9, 1909.

### Specific Therapeutics.

A recent article contributed to the *Times*, in which the writer summarises in more or less popular language the present position of specific immunisation as a branch of therapeutics, is a useful reminder of a great change which has come over the spirit of medicine within the last twenty years. Mankind has for ever been on the look-out for specific remedies and has ransacked nature in his search, but until lately with scanty success. Mercury and the iodides for syphilis, quinine for malaria, iron for chlorosis; these, one might say, were the sole items in the catalogue of true specific remedies for disease, always excepting the preventive inoculation of small-pox, and, much later, of cowpox vaccine, a practice, so to speak, born out of time, a piece of pure empiricism, amply justified, yet by many centuries in advance of the knowledge which might have been expected to produce it. But with Pasteur the horizon widened and the world of pathogenic micro-organisms came into view. The success of his preventive inoculations against hydrophobia, though the agent of the infection was and is an unsolved mystery, brought to light the immense potentialities of the infected organism as a manufactory of specific remedies against the infections to which it was subject. It would seem that Pasteur must be regarded as the father of specific immunisation. This one may say without in any way depreciating the immense debt owed by the world to Jenner. For the treatment which he exploited with such happy results was, as we have said, an isolated item of empirical medicine, prolific of benefits as became a wonder properly belonging to the nineteenth century yet born by a kind of happy accident in the eighteenth; barren in that men were as yet without the knowledge which would permit its applications to be extended. To this knowledge Pasteur gave us the key when he showed us the world of microbes; a master-key indeed, like every fresh generalisation which withstands the test of time and enlarged experience.

From the day when Pasteur demonstrated that the virus of rabies could be attenuated, and when thus attenuated could produce an immunity to virulent examples of the poison, we have been steadily advancing along the road of specific immunisation. Not without stumbles, it is true, and even serious falls. Our readers will remember the high hopes raised by Koch's introduction of the original tuberculin; how eagerly it was tried, and how sadly the profession gave its adverse verdict. But the set-back was only temporary, and, though for the time being disheartened, the believers in specific immunisation by no means despaired. Presently Von Behring's discovery of the diphtheria antitoxin and the rapid success it achieved gave a fresh stimulus to the work, and thenceforward serum and vaccine therapies took an assured position. The present methods of specific immunisation fall into two categories. In the one class come diseases such as diphtheria and tetanus, diseases in which the infection is essentially local, the symptoms depending to a variable but large extent upon the soluble poisons produced at the local manufactory, but exercising their baneful effects upon distant parts. For such diseases the agent required is an antitoxin—a strict and specific antidote to a chemical poison, which shall neutralise it in a way analogous to the action of acid and alkali upon one another. This is obtained, as is well known, by the inoculation of horses with the virus in question until the serum of the animal contains the specific antidote in quantity. This antidote-containing serum is then injected into the infected individual, the antidote combines with the specific poison circulating in the patient's blood, is neutralised and becomes inert. This process is known as "passive immunisation," the antidote having a source external to the patient's body. In the other category come diseases due to organisms whose poisons are less diffusible, and whose malign effects are exerted more strictly in the neighbourhood in which the infection is established; diseases in which the infective agent sometimes enters the blood-stream and multiplies in it or is carried to remote parts, and being deposited there commences its mischievous growth. Of these are tuberculosis and gonorrhœa. The poison of the organism being little or not at all diffusible it is clear that no mere antidote is likely to achieve success; but the local manufactory must be locally attacked by increasing the specific defences of the individual assailed, such as the phagocytic activity of his cells and the production of those "opsonic" qualities in the serum, which are held by Sir Almroth Wright and his school to dictate the extent to which phagocytosis can proceed. The method by which this is attempted consists in the inoculation of the affected person with killed cultures of the infecting organism. It will be seen that this method is precisely analogous to vaccination against smallpox, for its principle, like that of the latter, is the inoculation of an attenuated virus of the disease to be combated. In the case of smallpox the "vaccine" is the virus of the disease attenuated by passage through a calf; in the case of bacterial inoculations the vaccine is a virus attenuated by being killed, and the analogy is so close that the terms "vaccine" and "vaccination" are now no longer limited to the preventive treatment of smallpox. Thus a "gonococcal vaccine" is a killed culture of the gonococcus, a "bacillus coli vaccine" a killed culture of this organism. The increase in specific resistance thus conferred is spoken of as "active immunisation" because it depends upon the cellular activity of the infected tissues themselves.

Later experiences have shown that the disappointing results of Koch's original vaccine against tuberculosis were largely due to excessive dosage, and much effort has been expended in the attempt to provide a criterion by which the doses of vaccines may be regulated. The prime worker in this field is Sir Almroth Wright, the inventor of the "opsonic index" of which so much has been heard of late. The battle still raging round the virtues and failings of this method of control continues to keep counsel darkened. But it is necessary to observe that whether the "opsonic index" stands or falls, it offers a problem totally distinct from vaccination and its benefits. Vaccination with bacterial cultures, though still in its infancy, has taken a permanent place in therapeutics. The "opsonic" control is but ancillary to it and may fail to justify itself.

